# Development of genomic SSR markers for fingerprinting lettuce (*Lactuca sativa* L.) cultivars and mapping genes

**DOI:** 10.1186/1471-2229-13-11

**Published:** 2013-01-22

**Authors:** Gilda Rauscher, Ivan Simko

**Affiliations:** 1 United States Department of Agriculture, Agricultural Research Service, U.S. Agricultural Research Station, 1636 E. Alisal St, Salinas, CA, 93905, USA; 2 Present address: Agricultural Biotechnology, DuPont Pioneer, Wilmington, Wilmington, DE, 19880, USA

**Keywords:** Data resolution statistics, Genotyping, *Lactuca*, Linkage map, Marker distribution, Microsatellites

## Abstract

**Background:**

Lettuce (*Lactuca sativa* L.) is the major crop from the group of leafy vegetables. Several types of molecular markers were developed that are effectively used in lettuce breeding and genetic studies. However only a very limited number of microsattelite-based markers are publicly available. We have employed the method of enriched microsatellite libraries to develop 97 genomic SSR markers.

**Results:**

Testing of newly developed markers on a set of 36 *Lactuca* accession (33 *L*. *sativa*, and one of each *L*. *serriola* L., *L*. *saligna* L., and *L*. *virosa* L.) revealed that both the genetic heterozygosity (*UHe* = 0.56) and the number of loci per SSR (*Na* = 5.50) are significantly higher for genomic SSR markers than for previously developed EST-based SSR markers (*UHe* = 0.32, *Na* = 3.56). Fifty-four genomic SSR markers were placed on the molecular linkage map of lettuce. Distribution of markers in the genome appeared to be random, with the exception of possible cluster on linkage group 6. Any combination of 32 genomic SSRs was able to distinguish genotypes of all 36 accessions. Fourteen of newly developed SSR markers originate from fragments with high sequence similarity to resistance gene candidates (RGCs) and RGC pseudogenes. Analysis of molecular variance (AMOVA) of *L*. *sativa* accessions showed that approximately 3% of genetic diversity was within accessions, 79% among accessions, and 18% among horticultural types.

**Conclusions:**

The newly developed genomic SSR markers were added to the pool of previously developed EST-SSRs markers. These two types of SSR-based markers provide useful tools for lettuce cultivar fingerprinting, development of integrated molecular linkage maps, and mapping of genes.

## Background

Cultivated lettuce (*Lactuca sativa* L.) is a self-fertilizing diploid species from the family of Compositae (Asteraceae) with 2*n* = 2*x* = 18 chromosomes. Several horticultural types of lettuce are cultivated worldwide for human consumption. Classification of lettuce cultivars into horticultural types is generally based on head and leaf shape, size, and structure and stem length. The seven types include crisphead (combined iceberg and Batavia-type lettuces), romaine, butterhead, Latin, leaf, stem, and oil lettuces.

Several types of biochemical and molecular markers have been applied for lettuce genotyping, such as isozymes [1], restriction fragment length polymorphism - RFLP [2], random amplified polymorphic DNA - RAPD [3], amplified fragment length polymorphism - AFLP [4], simple sequence repeats - SSR [5], target region amplification polymorphism - TRAP [6,7], expressed sequence tag based SSR - EST-SSR [8], single nucleotide polymorphism – SNP [9], and single position polymorphism – SPP [10]. Genotyping with molecular markers is used for cultivar fingerprinting, detection of genetic diversity, assessment of population structure, mapping genes of interest, and for selection of desirable genotypes in breeding programs. Fingerprinting of plant cultivars is frequently carried out with SSR markers (microsatellites) because they are co-dominant, multi-allelic and thus more informative than dominant-types of markers. However, development of SSR markers is costly and time-consuming and therefore only a very limited number of SSR markers are publicly available for lettuce [5]. Previously, we have developed a set of EST-SSR markers [8] from approximately twenty thousand unigenes of *L*. *sativa* and its close wild relative prickly lettuce (*L*. *serriola* L.). In the present work we describe the development of SSR markers from genomic DNA for fingerprinting lettuce cultivars. To develop this set of novel SSR markers we used the method of enriched microsatellite libraries [11-13].

Objectives of the present work were to 1) develop a set of genomic SSR markers; 2) test marker polymorphism on a diverse set of lettuce cultivars; and 3) integrate the SSR markers into the molecular linkage map of lettuce.

## Methods

### Development of genomic SSR markers

Genomic SSR markers were developed from *L*. *sativa* cv. Salinas according to the protocols of Glenn and Schable [13] and Farias et al. [12], with some modifications. The procedure consists of DNA extraction, DNA digestion with a restriction enzyme, ligation of linkers to DNA fragments, PCR-enrichment for microsatellite-containing fragments, hybridization to microsatellite-specific probes, recovery of microsatellite-containing fragments, and cloning and sequencing of products.

Approximately 100 mg of tissue from young leaves of a month-old, greenhouse-grown plant was collected and immediately lyophilized. The sample was ground to fine powder using a TissueLyser mill before extracting DNA with DNeasy Plant Mini Kit (both from Qiagen, Valencia, CA). The DNA concentration and quality was analyzed with an ND-1000 Spectrometer (NanoDrop Technologies, Wilmington, DE). Three μg of genomic DNA was digested with *BfuCI*, an isoschizomer of *Sau*3AI (New England Biolabs Ipswich, MA) according to the manufacturer’s instructions. The enzyme was deactivated at 80°C for 20 min and a 5 μl aliquot was run on a 0.8% agarose gel to verify the digestion. The linkers were created by hybridizing two oligonucleotides: Er1BhGATCSticky 5^′^-GAT CGG CAG GAT CCA CTG AAT TCG C-3^′^ and Er1Bh1Blunt 5^′^-GCG AAT TCA GTG GAT CCT GCC-3^′^. These linkers were then ligated to the fractioned DNA using T4 DNA ligase (New England Biolabs, Ipswich, MA) following the manufacturer’s instructions.

A PCR was set up to increase quantity of the fragments that are containing SSRs. PCR-enrichment was performed using the product of the ligation as a template and Er1Bh1Blunt as a primer. The reaction was set up as follows: 1 × PCR ready mix (Promega, Fitchburg, WI), 0.25 mM Er1BhBlunt primer, 1 μl template and bidistilled water to 25 μl final volume (Table 1). Unincorporated nucleotides and primers were cleaned up with Exonuclease I and Antarctic phosphatase (New England Biolabs, Ipswich, MA). The oligonucleotide probes were biotinylated using terminal transferase (New England Biolabs, Ipswich, MA) following the manufacturer’s instructions. In order to produce 1–3 biotins per oligonucleotide, a proportion of 1 pmol of 3^′^ ends to 0.01 mmol of biotin-14-dATP (Invitrogen, Grand Island, NY) was used. The oligonucleotides were mixed as suggested by Glenn and Schable [13]: mix 2 ((AG)_12_, (TG)_12_, (AAC)_6_, (AAG)_8_, (AAT)_12_, (ACT)_12_, (ATC)_8_); mix 3 ((AAAC)_6_, (AAAG)_6_, (AATC)_6_, (AATG)_6_, (ACAG)_6_, (ACCT)_6_, (ACTC)_6_, (ACTG)_6_); and mix 4 ((AAAT)_8_, (AACT)_8_, (AAGT)_8_, (ACAT)_8_, (AGAT)_8_). The mixes were biotinylated independently at 37°C for 30 min and the enzyme was deactivated by heating to 70°C for 10 min. The excess biotin was removed using precipitation with 3 M sodium acetate and absolute ethanol, and resuspending the probes in 100 μl of bidistilled water. To isolate SSR-containing fragments, the probes were attached to Streptavidin magnetic beads (New England Biolabs, Ipswitch, MA) according to the manufacturer’s instructions. The product of the enrichment-PCR was denatured at 95°C for 5 min and quickly chilled on ice. This product was then hybridized to the probes in an oven at 55°C for 3 hours and washed with 2 × SSC buffer and 0.1% SDS buffer twice, and then with 1 × TE buffer-50 mM NaCl and resuspended in 200 μl of 1 × TE buffer. To recover SSR-containing fragments, the probe-SSR complex was denatured at 95°C for 5 min and the beads were quickly removed with a magnet. A PCR was set up to test recovery of fragments using the Er1Bh1Blunt oligonucleotide as a primer and the product of the hybridization as a template (Table 1). The PCR products were then run on a 1.2% agarose gel.

**Table 1:** PCR conditions

**PCR purpose**	**PCR conditions (initial denaturation, number of cycles, denaturation, annealing, elongation, and a final extension step)**
Enrichment for microsatellite-containing fragments	94°C for 2 min, followed by 12 cycles of 94°C for 15 sec, 55°C for 35 sec, 72°C for 90 sec
Recovery of microsatellite-containing fragments	35 cycles of 94°C for 15 sec, 55°C for 35 sec, 72°C for 30 sec, and a final extension at 72°C for 5 min
Preparation of products for cloning	94°C for 2 min, followed by 15 cycles of 94°C for 15 sec, 55°C for 35 sec, 72°C for 30 sec, and a final extension at 72°C for 5-10 min
Confirmation of cloned products	96°C for 2 min, followed by 33 cycles of 94°C for 40 sec, 57°C for 12 sec, 72°C for 30 sec, and a final extension at 72°C for 5 min
Genotyping with SSRs	96°C for 2 min, followed by 33 cycles at 94°C for 35 sec, annealing temperature* for 15 sec, 72°C for 30 sec, and a final extension at 72°C for 5 min

* Annealing temperatures for each SSR are shown in Additional file 1.

Once the fragment recovery was verified, a second PCR-enrichment was set up to prepare sequences for cloning. Four reactions were set up with 0.8 mM dNTPs, 1× PCR buffer, 0.4 μM Er1Bh1Blunt primer, 2.5 U Taq Polymerase and 1 μl of the hybridization product (Table 1). The PCR products were pooled, cleaned with QiaQuick columns (Qiagen, Valencia, CA), and cloned using Topo TA cloning kit for sequencing and *E*. *coli* Mach1-T1^R^ cells (Invitrogen, Grand Island, NY), according to the manufacturer’s instructions. Transformed cells were passed to 96 well plates with lysogeny broth (LB) containing 50 mg/ml ampicillin, and grown for at least 4 hours at 37°C. A confirmation PCR was carried out using standard M13 forward and reverse primers and 2–3 μl of the LB medium with bacterial growth as a template. Bovine serum albumin in the concentration of 25 μg/ml was added to the PCR; all other reagents were used in concentrations described above. *E*. *coli* colonies that contained products of expected size were transferred to Wu Broth supplemented with ampicillin and submitted for sequencing to the USDA-ARS Genomics and Bioinformatics Research Unit in Stoneville, MS. Sequencing data were cleaned up from vector contamination and assembled in contigs using CLC DNA workbench 5.0 (CLCBio Aarhus, Denmark). The SSRs with the minimal length of 14 bp were identified using WebSat [14]. Primers for SSR amplification were designed by Primer3 software [15] integrated into WebSat. Primer quality analysis was performed with OligoAnalizer 3.1 (Integrated DNA Technologies Inc, Coralville, IA). When sequences contained multiple SSRs, different primer-pairs were designed for each SSR. If amplification with the Primer 3-designed primers did not yield expected products, a second pair of primers was designed using CLC DNA workbench. Sequences of SSR-containing fragments were compared in January 2012 to the GenBank database (http://www.ncbi.nlm.nih.gov) using CLC DNA workbench 5.0. The ‘blastn’ option of the BLAST algorithm [16] was applied to search the nucleotide collection (nr) of the viridiplantae database using low complexity filter to avoid spurious hits based on microsatellite sequence only. The threshold of significance to report similarity was set at 1e-4.

### Testing of marker polymorphism

A set of 36 accessions was used to test polymorphism of newly developed SSR markers. This set comprised 33 *L*. *sativa* cultivars plus a single accession from each of the three wild species sexually compatible with cultivated lettuce; prickly lettuce (*L*. *serriola* L.), willowleaf lettuce (*L*. *saligna* L.), and bitter lettuce (*L*. *virosa* L.). Genotyped cultivars belonged to seven horticultural types: crisphead, leaf, romaine, butterhead, stem, Latin, and oil lettuce (Table 2).

**Table 2:** **List of 36 *Lactuca *accessions genotyped with genomic SSR markers**

**Horticultural type or species**	**Accession**
Butterhead	Bibb, Big Boston, Dark Green Boston, Margarita
Crisp	Calmar, Empire, Great Lakes 54, Iceberg, La Brillante, Reine des Glaces, Salinas, Salinas 88, Vanguard, Winterhaven
Latin	Eruption, Little Gem
Leaf	Australian, Grand Rapids, Lolla Rossa, Prizehead, Red Oak Leaf, Red Salad Bowl, Salad Bowl
Oil	PI 251246
Romaine	Clemente, Heart’s Delight, Paris Island Cos, PI 207490, Triple Threat, Valmaine
Stem	Balady Aswan, Celtuce, Da Ye Wo Sun
*L*. *saligna*	PI 509525
*L*. *serriola*	UC96US23
*L*. *virosa*	IVT 280

Genotyping with SSR markers: The PCR conditions for SSR amplification were optimized for each primer pair. The optimal PCR conditions are described in Additional file 1. In general, the reactions were set up using 0.2 μM of each primer, 5 ng of DNA template, and 1× of Taq PCR master mix (New England Biolabs, Ipswich, MA) in a final volume of 10 μl (Table 1). The PCR products were separated using eGene HDA-GT12 DNA analyzer (currently known as QIAxcel System, from Qiagen, Valencia, CA) and scored by Biocalculator software (eGene, Irvine, CA).

Analysis of genetic heterozygosity: The statistical analyses of SSR data were performed with GenAlEx 6.1 [17] for codominant markers and GenoDive v.2.0b20 [18]. Missing data and null alleles were excluded from the analysis. The unbiased estimate of genetic heterozygosity *UHe*[19] and observed number of different alleles *Na* were used to measure marker informative value (GenAlEx 6.1). Genetic distances (*F*_*st*_) [20] between all pairs of horticultural types with at least two accessions, analysis of molecular variance (AMOVA) [21], and principal components analysis (PCA) were calculated using GenoDive v.2.0b20. The significance of the differences between the EST-based [22] and genomic SSRs were tested with Student’s *t*-test.

Consistency of molecular marker datasets: Data resolution (DR) statistics were used to evaluate the internal consistency of the SSRs dataset with the program written by van Hintum [23]. DR values can be in the range from 0 to 1; where higher values indicate higher internal consistency of the data. The number of replications was set to 10,000.

Identification of genotypes: The software MultiLocus ver. 1.3b [24] was used to estimate the number of different genotypes that can be identified in a set of 36 accessions with a gradually increasing number of markers. This analysis shows whether scoring more markers leads to increasing number of identified genotypes. One thousand samplings of markers were performed at random from 1 to *m*-1, where *m* is the total number of markers. The relative number of identified genotypes was calculated by dividing the number of identified genotypes by the total number of accessions.

### Integrating SSR markers into the molecular linkage map of lettuce

Newly developed genomic SSRs were integrated into the *L*. *sativa* (cv. Salinas) × *L*. *serriola* (accession UC96US23) molecular linkage map [25]. A framework linkage map consisted of SNP and AFLP markers spaced approximately 5–10 cM apart and covering all nine lettuce linkage groups. These framework markers were selected from the integrated SNP/AFLP linkage map, and the marker information was downloaded in April 2010 from the Compositae Genome Project website (compgenomics.ucdavis.edu/compositae_LettMap.php). Both parental genotypes and 96 F_8_ recombinant inbred lines (RILs) from the interspecific *L*. *sativa* × *L*. *serriola* mapping population were genotyped with SSRs. DNA isolation and genotyping with SSRs was carried out as described above. Integration of the SSR markers into the framework linkage map was performed using MapManager QTX version 0.30 [26]. Program settings included SelfRI for linkage evaluation, the Kosambi mapping function, inference of missing data, and the command for marker distribution with *p*-value ≤ 0.001. In addition to genomic SSRs, EST-SSR markers previously developed in our laboratory [22] were also integrated into this molecular linkage map.

### Modeling and analysis of marker distribution

To analyze distribution of the SSR markers on the molecular linkage map, we compared the observed distribution of markers with a model that assumes a random distribution of markers. This model was developed by randomly placing markers on nine linkage groups of the molecular linkage map. One thousand models were generated for each linkage group populated with genomic SSRs and EST-SSRs. Analyses of marker distribution were based on 1) the length of intervals between two successive markers and 2) the clustering of markers. The length of intervals (in cM) between two successive markers was calculated from the linkage map (or modeled data). Subsequently, the intervals were grouped into bins containing intervals of similar size (in 10 cM increase). Evaluation of clustering was carried out by dividing each of the nine linkage groups into segments 20 cM long. The number of markers per 20 cM-long segment was counted for both the real and modeled data.

Goodness of fit between observed and modeled distributions of markers was analyzed both with Kolmogorov–Smirnov (K-S) test, and the Pearson’s Chi-square (*χ*^2^) test. Modeling and statistical analyses were performed with Microsoft Excel v.14.1.4 (Microsoft, Redmond, WA) and JMP 6.0.3 (SAS Institute, Cary, NC, USA).

## Results and discussion

### Marker development

A total of 217 products were amplified from 548 bacterial colonies grown on a selective media (LB with ampicillin). One hundred and fifty-four of these products originated from mix 2, 24 from mix 3, and 39 from mix 4. Out of 217 products, 192 were sequenced, yielding 117 unique sequences that contained microsatellites. Sequencing revealed that some fragments contain more than one microsatellite. In such case, an attempt was made to design primers that would amplify each microsatellite individually. The microsatellite-containing sequences were named based on their origin (*Lactucasativa* cv. Salinas) followed by a plate code (A or B) and a consecutive number (LSSA ## or LSSB ##).

Seventy-nine percent of sequenced products contained dinucleotide repeats; 14% of products contained trinucleotide repeats; 3% of products contained tetranucleotide repeats, and 4% of products contained repeats consisting of five or more nucleotides. Two separate repeats were detected in 24% of products and imperfect repeats were found in 22% of products.

### Results of sequence homology searches

**Figure 1:**
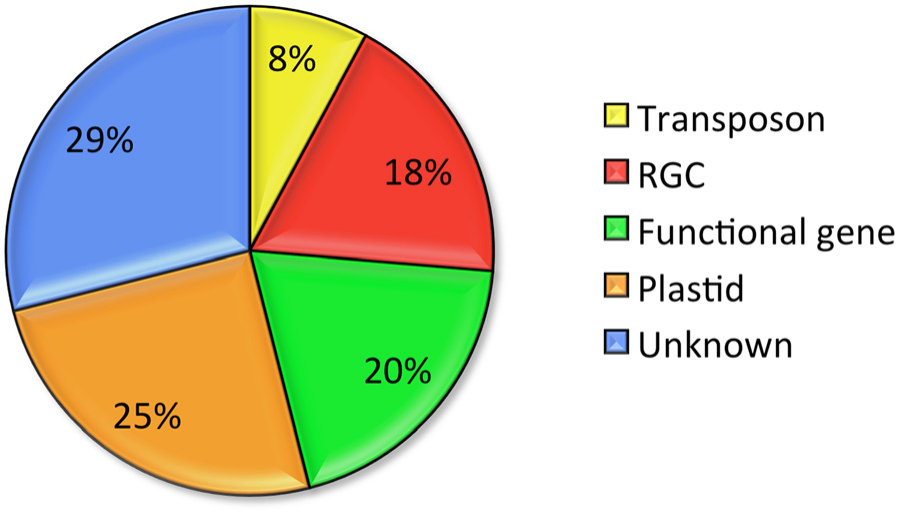
**Homology of sequences containing microsattelites**. Transposon group contains sequences similar to transposable elements (both transposons or retrotransposons). RGC group contains sequences similar to resistance gene candidates or RGC pseudogenes. Functional gene group contains sequences that are similar to proteins with known function, but not those in the RGC group. Plastid group includes sequences similar to chloroplast or mitochondrion.

Seventy-six SSR-containing fragments showed high sequence similarity (<1e-4) to the nucleotide collection of the viridiplantae GenBank database (http://www.ncbi.nlm.nih.gov). Nineteen sequences were similar to chloroplast DNA (cpDNA) or mitochondrion DNA (mtDNA). However, several of these sequenced fragments showed segregation in the mapping population, indicating that they are likely originating from a nuclear DNA having sequence similarity to cpDNA or mtDNA. In plant species, a large percentage (61.4% to 94.3%) of mtDNA sequence is highly similar to nuclear DNA sequences [27]. A group of 14 sequenced fragments appeared to be highly similar to resistance gene candidates (RGCs) and RGC pseudogenes [28]. The SSR markers developed from these fragments can be evaluated for association with disease resistance and (if association is detected) possibly used in tagging resistance phenotypes. Six of the sequenced fragments were similar to transposons or retrotransposons. Fifteen sequences matched to a number of putative genes including genes for chitinase, peroxidase, trypsin inhibitor, kaurene oxidase, or teosinte branching. The remaining 22 fragments did not show significant similarity to the genes or putative genes with known function (Figure 1).

### Marker informative value and analysis of accessions

**Figure 2:**
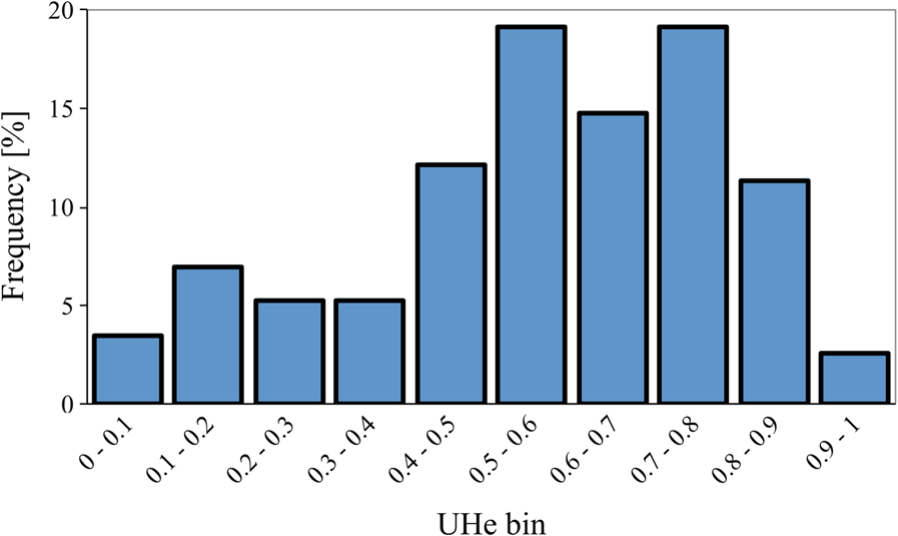
**Distribution of unbiased estimate of genetic heterozygosity (*UHe*) for 97 genomic SSRs**. The mean *UHe* value for SSRs is 0.56.

When developed SSR-markers were used for genotyping the set of 36 accessions, several markers had sizes substantially longer than was expected, showed several loci per homozygous accessions, or were amplified only in very few accessions. These markers were excluded from further analyses, reducing the number of good quality markers to 97 (Additional file 1). Genetic heterozygosity, as measured by unbiased estimate *UHe*, ranged from 0 (for monomorphic markers) to 0.92 with the mean value of 0.56 (Figure 2). The average *UHe* for genomic SSR markers was significantly higher (*t*-test, *p* = 5.9 × 10^-11^) than *UHe* observed on EST-SSR (0.32) [22]. Similarly, the number of loci per SSR (*Na*) was significantly higher (*t*-test, *p* = 7.3 × 10^-6^) for genomic-derived markers than for the EST-SSRs. The number of loci per genomic SSR ranged from 1 to 19 with the mean value of 5.50; while the mean value for EST-SSRs was 3.56 [22]. Though the set of accessions previously genotyped with EST-SSRs [22] is not identical with the current set of accessions genotyped with genomic SSRs, the two sets overlap. Both sets contain material from the same horticultural types of lettuce allowing a limited comparison. The lower polymorphism of EST-SSR markers as compared with genomic SSRs has been reported in several other plant species, such as grape [29], rice [30], wheat [31], and sunflower [32]. The lower polymorphism in EST-SSRs is likely due to the conserved nature of coding regions of a genome [33].

**Figure 3:**
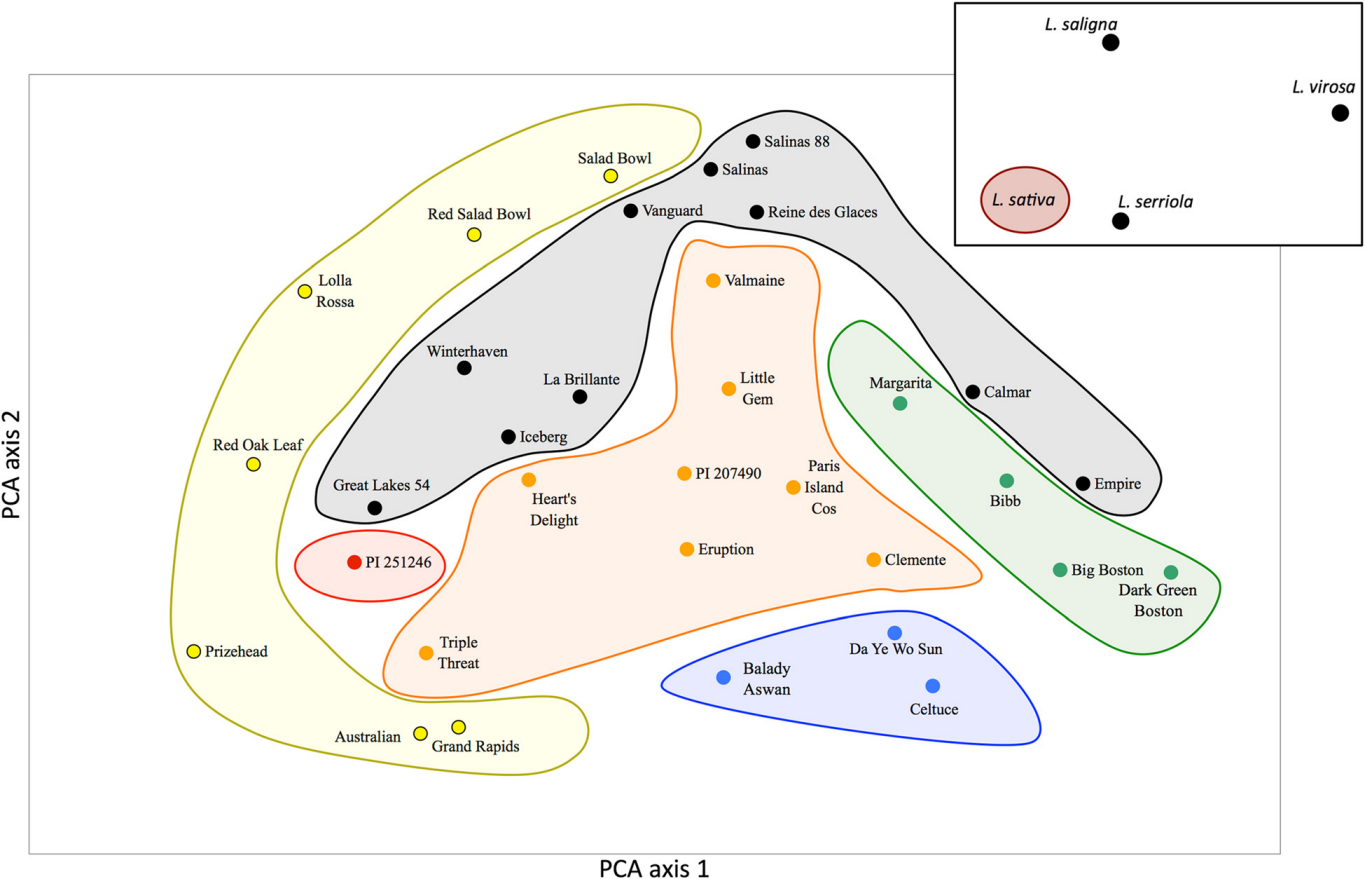
**Principal component analysis (PCA) of the 33** ***L*. *sativa *accessions and three wild species genotyped with 97 genomic SSRs.** Color coding for horticultural types is: leaf – yellow, crisp – black, oil – red, romaine and Latin combined – orange, stem – blue, butterhead – green. Insert in the upper right corner shows the relative position of *L*. *serriola* (UC96US23), *L*. *saligna* (PI 509525), and *L*. *virosa* (IVT 280) to the set of *L*. *sativa* accessions.

Results from AMOVA indicate that approximately 3.2% of genetic diversity was within accessions, 78.9% (*p* < 0.001) among accessions, and remaining the 17.9% (*p* < 0.005) among horticultural types (Table 3). These results are similar to those achieved with SNP markers that were used to genotype five horticultural types of lettuce. Kwon et al. [9] detected that 23% of the genetic variation resided among horticultural types, while 68.2% resided within horticultural types. We also calculated pairwise differentiation (*F*_*st*_) for all pairs of horticultural types with at least two accessions per type (Table 4). The variation in the *F*_*st*_ values ranged from 0.038 (between crisp and romaine types) to 0.202 (between butterhead and leaf types). These results were different from our previous analyses with TRAP [7] and EST-SSR markers [22], which separated crisp and romaine types into respective subpopulations [7] or clusters [22]. The results of PCA revealed that accessions of some horticultural types clustered together (e.g. stem lettuces) and were well separated from other types, while accessions from some other types did not cluster well (Figure 3). For example crisp-lettuce accessions appear to form three separate sub-clusters, one containing four accessions (Great Lakes, Winterhaven, La Brillante, and Iceberg), the second consisting of Vanguard, Salinas, Salinas 88, and Batavia Reine des Glaces, and the third group of two accessions (Calmar and Empire). Because of this distribution of accessions, *F*_*st*_ values for crisp type are generally low and range from only 0.038 (with romaine lettuce) to 0.145 (with stem lettuces). Though PCA unambiguously separated all wild species from cultivated lettuce, *L*. *serriola* was closest to the cluster of *L*. *sativa* accessions, while *L*. *virosa* was the most distant from this cluster (Figure 3, insert in the upper right corner). The observed distance between wild species and *L*. *sativa* accessions corresponds to sexual compatibility of the three species with cultivated lettuce. Similarly, the marker transfer rate was highest to *L*. *serriola* (83%) that is the closest relative of cultivated lettuce, followed by *L*. *saligna* (66%) and *L*. *virosa* (63%). These results correspond to previous observations obtained with both genomic SSRs and EST-SSRs [22].

**Table 3:** Analysis of molecular variance (AMOVA) calculated from genomic SSR markers

**Source of variation**	**Percentage of variation**	***p*-value**
Within accessions	3.2	
Among accessions	78.9	< 0.001
Among horticultural types	17.9	< 0.005

**Table 4:** **Pairwise differentiation (*F***_***st***_**) among horticultural types calculated from genomic SSR markers**

**Horticultural type**	**Crisp**	**Butterhead**	**Latin**	**Leaf**	**Romaine**	**Stem**
Crisp	-	0.105	0.048	0.096	0.038	0.145
Butterhead		-	0.178	0.202	0.134	0.164
Latin			-	0.084	0.060	0.117
Leaf				-	0.101	0.157
Romaine					-	0.136
Stem						-

### Consistency of datasets and genotypic diversity

**Figure 4:**
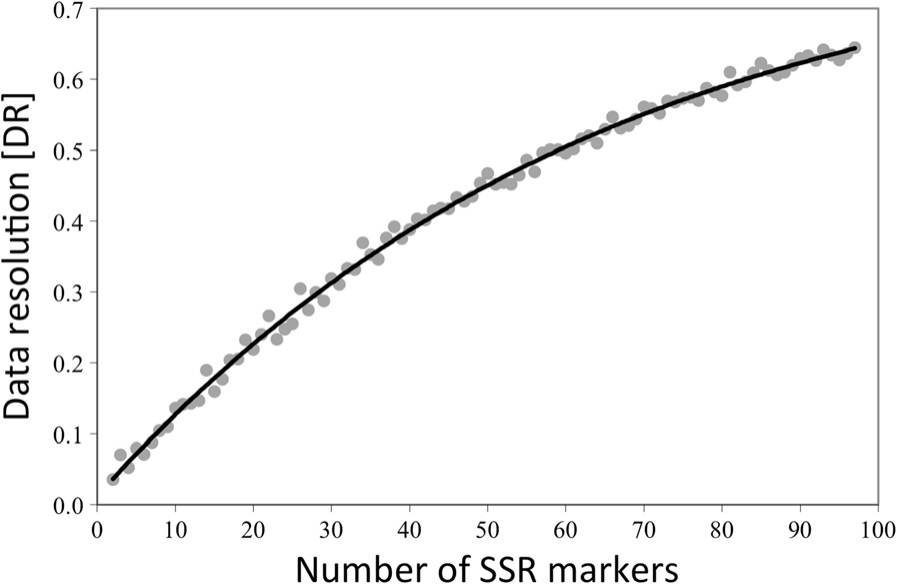
**Data resolution (DR) curve for the 97 genomic SSR markers**. The minimum DR value of two markers is 0.036; the maximum DR value of 97 SSR markers is 0.644.

DR analysis shows the expected shape of the curve with a low initial value of 0.036 for two markers and gradual increase to the value of 0.644 for 97 markers (Figure 4). The higher DR values indicate the higher internal consistency of the SSR dataset when more markers are analyzed. Van Hintum [23] observed similar results for *L*. *serriola* accessions genotyped with AFLP markers. In our analyses, 61 SSR markers were needed to reach the DR of 0.5; an estimate from Van Hintum [23] indicates that approximately 70 AFLP markers were needed to reach the same DR value. It was previously observed that for the same number of markers, consistency of SSR datasets is usually higher than consistency of datasets of dominant markers [23,34].

**Figure 5:**
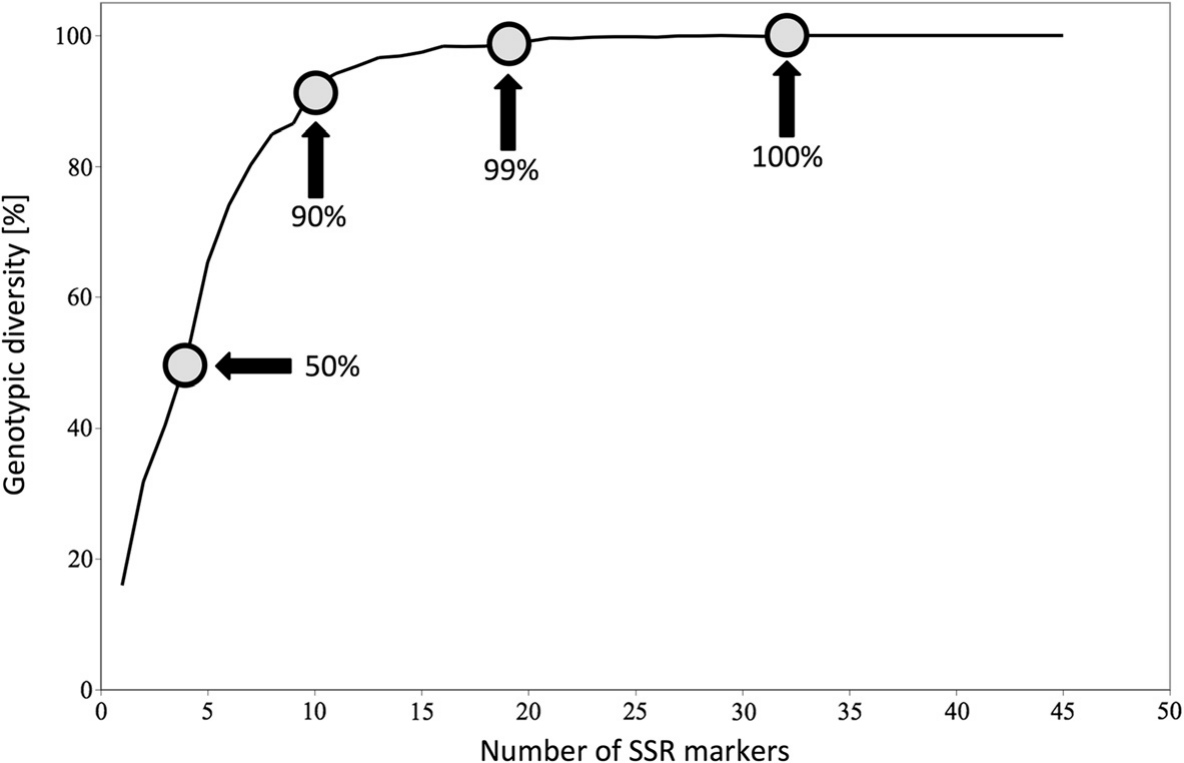
**Effect of the increasing number of genomic SSR markers on the estimate of genotyping diversity.** Circles indicate genomic diversity of 50%, 90%, 99%, and 100%, respectively. The value of 100% was reached with 32 and more markers.

To analyze whether scoring more SSR markers increases likelihood of distinguishing more genotypes, the genotypic diversity analysis was performed on the set of 36 accessions. On average only four markers were needed to identify 50% of genotypes, 10 markers were needed to identify 90% of genotypes, and 19 markers were needed to identify 99% of genotypes (Figure 5). Our analysis shows that any 32 SSR markers were able to distinguish genotypes of all 36 accessions unambiguously. This is a relatively high number of markers that are needed for genotyping. For example, only 17 SSR markers on average were required to identify 54 sugar beet hybrid varieties [34]. However, variability within certain horticultural types of lettuce is generally very low and similarity among accessions of the same type is high [7,22]. Therefore more molecular markers are needed to distinguish closely related material with high genetic similarity. In addition, some of the SSRs tested in the present study originate from the same genomic region, thus limiting their ability to distinquish genotypes.

### Distribution and clustering of SSR markers on the interspecific molecular linkage map

**Figure 6:**
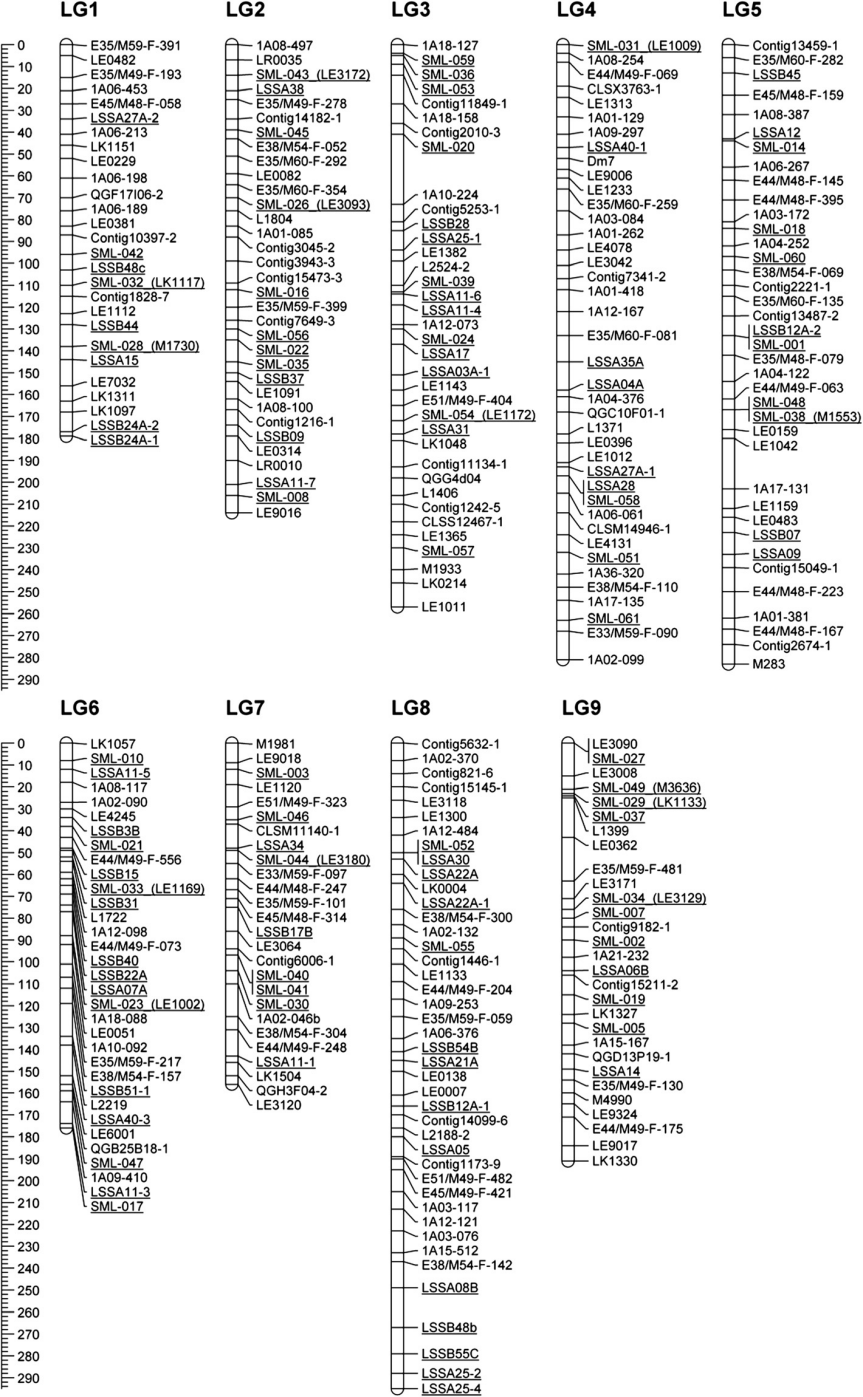
**Distribution of microsatellite markers on the molecular linkage map of lettuce**. The framework linkage map was based on the segregation of alleles in the *L*. *sativa* (cv. Salinas) × *L*. *serriola* (accession UC96US23) mapping population [25]. EST-SSR markers [22] are named SML; while genomic SSR markers are named LSSA or LSSB. Scale for the linkage map is indicated on the left side in cM. SSR-based markers are underlined.

We mapped 54 genomic SSR markers on the molecular linkage map of lettuce that was based on the segregation of alleles observed in the Salinas (*L*. *sativa*) × UC96US23 (*L*. *serriola*) mapping population (Figure 6). Remaining SSRs were not mapped due to homozygosity between the parents, or due to weak linkage with markers on the framework map. The mapped SSR markers were distributed on all nine linkage groups (LG). The fewest markers were located on LG 9 (two markers) and the most markers were located on LG 8 (12 markers). The goodness of fit test indicates that the distribution of markers on linkage groups was not significantly different from the even distribution of markers (*p* = 0.115). In addition to genomic SSRs, we have also mapped 52 previously developed EST-SSRs [22], bringing the combined total number of mapped SSRs to 106. Interestingly, the fewest EST-SSRs were located on LG 8 (two markers) that harbors the highest number of genomic SSRs (12 markers). However, a difference in the distribution of genomic SSRs and EST-SSRs over all LGs was not significant (*p* = 0.056). Similarly as with genomic SSRs, distributions of EST-SSRs and a combined group of genomic and EST-SSR markers were not significantly different from the even distribution of markers over all LGs (*p* = 0.422, and *p* = 0.770, respectively). Truco et al. [25] reported 729 AFLP and 18 SSR markers in the same mapping population. The highest number of markers (142) were reported on LG 4, while the fewest markers (51) were observed on LG 9.

**Figure 7:**
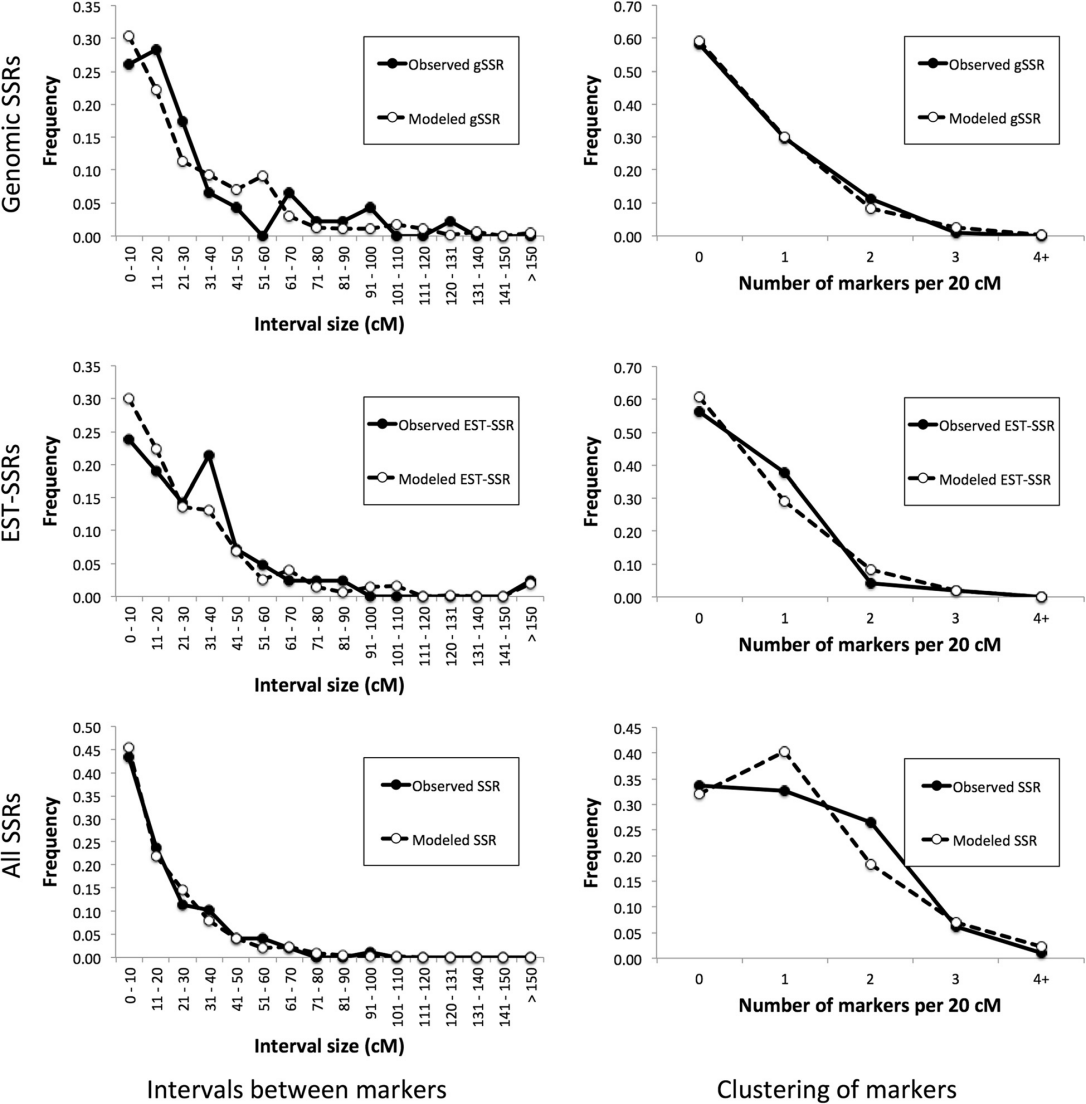
**Length of intervals between two successive SSR markers (left column) and the number of SSR markers per 20 cM-long segment (right column).** Information is shown for genomic SSRs (top row), EST-SSRs (middle row), and a combined group of genomic SSR and EST-SSR markers (bottom row). Observed data are indicated by full circle and solid line, modeled data based on a random distribution of markers are indicated by open circle and dashed line.

The average length of intervals between two successive markers was 29 cM for genomic SSRs, 30 cM for EST-SSRs, and 18 cM when all mapped SSRs were considered. The genome-wide distribution of the length of intervals matches well with the modeled distribution (Figure 7, left column). Neither the K-S test nor the *χ*^2^ goodness of fit test detected a significant difference between the modeled and the observed distribution of intervals (*p* values ranged from 0.502 to 0.823). Similarly, the observed number of markers per 20 cM-long segments matched well with the modeled data based on a random distribution of markers (Figure 7, right column). The *p*-values for the goodness of fit tests between the observed and the modeled data distribution ranged from 0.586 to 0.960 for the K-S test, and from 0.203 to 0.738 for the *χ*^2^ test. We observed that the modeled clustering of markers closely corresponds to a theoretical clustering based on the Poisson distribution (correlation of *r* = 0.999 for genomic SSRs, *r* = 0.995 for EST-SSRs, and *r* = 0.993 for a combined group of genomic and EST-SSR markers). Therefore the Poisson distribution can be used to identify parts of the linkage map where clustering of markers is higher than expected [35]. Using the Poisson distribution formula $p\left(k,\lambda \right)=\frac{e^{-\lambda }\lambda^{k}}{k!}$, where *k* (*k* = 0, 1, 2, 3, …) is the number of markers per segment for which a probability is being calculated, and λ is the mean number of markers per segment, we calculated that clustering of markers is suspected (at *p* < 0.01) if a 20 cM-long segment harbors five or more markers (when only genomic SSRs or only EST-SSRs are considered individually, four or more markers per segment indicate a possible clustering). Examination of the molecular linkage map revealed a single region with a possible clustering of markers. This cluster is located on LG 6, where nine markers (six genomic SSRs and three EST-SSRs) are located within the ~44 cM-long interval between markers LSSB3B and SML-023. Our results are in line with previous studies showing that the distribution of SSRs is usually even; though some clustering of markers (mainly around centromeric regions) is possible [36,37]. In lettuce, clustering of molecular markers in multiple genomic regions was previously reported for AFLPs, while only a few regions exhibited clustering of RFLP and RAPD markers [25].

## Conclusions

We have developed a set of 97 genomic SSRs and placed 54 of them on the interspecific molecular linkage map of lettuce. The SSR markers appear to be mostly randomly distributed in the genome with a possible cluster of markers in a single region on LG 6. Based on a sample of genotyping results, the maximum estimated genotyping error per sample is up to 8%. The highest error rate was observed when a difference in the size of analyzed alleles is below 3 bp. This rate of error is similar to that reported on maize [38], though it is higher than in some other reports [39,40]. Generally, genotyping of lettuce with genomic SSRs produces a higher error rate than genotyping with EST-SSR [22]. The factors increasing error rate involve a presence of stutter bands, high number of alleles per locus, and large product size [39]. The other possibility for a relatively high error rate observed in our genotyping system is that eGene DNA analyzer has a lower resolution than some other instruments used for SSR analysis [41]. The newly developed set of genomic SSRs in combination with previously developed EST-SSRs will be useful for cultivar fingerprinting, construction of integrated molecular linkage maps, and mapping genes of interest [42].

### Data access

Described sequences have been submitted to GenBank database under accession numbers JX474909 to JX474987.

## Abbreviations

AMOVA: Analysis of molecular variance; bp: Base pair; cM: Centimorgan; DR: Data resolution; EST: Expressed sequence tag; K-S test: Kolmogorov-Smirnov test; LB: Lysogeny broth; LG: Linkage group; Na: Number of loci per SSR marker; PCA: Principal component analysis; PCR: Polymerase chain reaction; RGC: Resistance gene candidates; SSR: Simple sequence repeat; UHe: Unbiased estimate of genetic heterozygosity.

## Competing interests

The authors declare that they have no competing interests.

## Authors’ contributions

GR and IS designed the study, GR performed laboratory experiments and sequence analyses, IS carried out modeling and marker data analyses, and GR and IS prepared the manuscript. Both authors read and approved the final manuscript.

## Supplementary Material

Additional file 1 **Primer combinations and PCR conditions for amplified genomic SSR markers.** Note that some primer pairs amplify two different microsatellites from the same sequenced product. Sequences with imperfect repeats are indicated by asterisk (*). Click here for file
